# A Review of Image Processing Techniques for Deepfakes

**DOI:** 10.3390/s22124556

**Published:** 2022-06-16

**Authors:** Hina Fatima Shahzad, Furqan Rustam, Emmanuel Soriano Flores, Juan Luís Vidal Mazón, Isabel de la Torre Diez, Imran Ashraf

**Affiliations:** 1 Department of Computer Science, Khwaja Fareed University of Engineering and Information Technology, Rahim Yar Khan 64200, Pakistan; hinafatimashahzad@gmail.com; 2 Department of Software Engineering, University of Management and Technology, Lahore 544700, Pakistan; furqan.rustam1@gmail.com; 3 Higher Polytechnic School, Universidad Europea del Atlántico (UNEATLANTICO), Isabel Torres 21, 39011 Santander, Spain; emmanuel.soriano@uneatlantico.es (E.S.F.); juanluis.vidal@uneatlantico.es (J.L.V.M.); 4 Department of Project Management, Universidad Internacional Iberoamericana (UNINI-MX), Campeche 24560, Mexico; 5 Project Department, Universidade Internacional do Cuanza, Municipio do Kuito, Bairro Sede, EN250, Bié, Angola; 6 Department of Signal Theory and Communications and Telematic Engineering, University of Valladolid, Paseo de Belén 15, 47011 Valladolid, Spain; 7 Department of Information and Communication Engineering, Yeungnam University, Gyeongsan 38541, Korea

**Keywords:** image processing, deep learning, video altering, deepfake

## Abstract

Deep learning is used to address a wide range of challenging issues including large data analysis, image processing, object detection, and autonomous control. In the same way, deep learning techniques are also used to develop software and techniques that pose a danger to privacy, democracy, and national security. Fake content in the form of images and videos using digital manipulation with artificial intelligence (AI) approaches has become widespread during the past few years. Deepfakes, in the form of audio, images, and videos, have become a major concern during the past few years. Complemented by artificial intelligence, deepfakes swap the face of one person with the other and generate hyper-realistic videos. Accompanying the speed of social media, deepfakes can immediately reach millions of people and can be very dangerous to make fake news, hoaxes, and fraud. Besides the well-known movie stars, politicians have been victims of deepfakes in the past, especially US presidents Barak Obama and Donald Trump, however, the public at large can be the target of deepfakes. To overcome the challenge of deepfake identification and mitigate its impact, large efforts have been carried out to devise novel methods to detect face manipulation. This study also discusses how to counter the threats from deepfake technology and alleviate its impact. The outcomes recommend that despite a serious threat to society, business, and political institutions, they can be combated through appropriate policies, regulation, individual actions, training, and education. In addition, the evolution of technology is desired for deepfake identification, content authentication, and deepfake prevention. Different studies have performed deepfake detection using machine learning and deep learning techniques such as support vector machine, random forest, multilayer perceptron, k-nearest neighbors, convolutional neural networks with and without long short-term memory, and other similar models. This study aims to highlight the recent research in deepfake images and video detection, such as deepfake creation, various detection algorithms on self-made datasets, and existing benchmark datasets.

## 1. Introduction

Fake content in the form of images and videos using digital manipulation with artificial intelligence (AI) approaches has become widespread during the past few years. Deepfakes, a hybrid form of deep learning and fake material, include swapping the face of one human with that of a targeted person in a picture or video and making content to mislead people into believing the targeted person has said words that were said by another person. Facial expression modification or face-swapping on images and videos is known as deepfake [1]. In particular, deepfake videos where the face of one person is swapped with the face of another person have been regarded as a public concern and threat. Rapidly growing advanced technology has made it simple to make very realistic videos and images by replacing faces that make it very hard to find the manipulation traces [2]. Deepfakes use AI to combine, merge, replace, or impose images or videos for making fraudulent videos that look real and authentic [3]. Face-swap apps such as ’FakeApp’ and ’DeepFaceLab’ make it easy for people to use them for malicious purposes by creating deepfakes for a variety of unethical purposes. Both privacy and national security are put at risk by these technologies as they have the potential to be exploited in cyberattacks. In the past two years, the face-swap issue has attracted a lot of attention, especially with the development of deepfake technology, which uses deep learning techniques to edit pictures and videos. Using autoencoders [4] and generative adversarial networks (GANs) [5], the deepfake algorithms may swap original faces with fake faces and generate a new video with fake faces. With the first deepfake video appearing in 2017 when a Reddit user transposed celebrities’ faces in porn videos, multiple techniques for generating and detecting deepfake videos have been developed.

Although deepfake technology can be utilized for constructive purposes such as filming and virtual reality applications, it has the potential to be used for destructive purposes [6,7,8,9]. Manipulation of faces in pictures or films is a serious problem that threatens global security. Faces are essential in human interactions as well as biometric-based human authentication and identity services. As a result, convincing changes in faces have the potential to undermine security applications and digital communications [10]. Deepfake technology is used to make several kinds of videos such as funny or pornographic videos of a person involving the voice and video of a person without any authorized use [11,12]. The dangerous aspect of deepfakes is their scale, scope, and access which allows anyone with a single computer to create bogus videos that look genuine [12]. Deepfakes may be used for a wide variety of purposes, including creating fake porn videos of famous people, disseminating fake news, impersonating politicians, and committing financial fraud [13,14,15]. Although the initial deepfakes focused on politicians, actresses, leaders, entertainers, and comedians for making porn videos [16], deepfakes pose a real threat concerning their use for bullying, revenge porn, terrorist propaganda, blackmail, misleading information, and market manipulation [3].

Thanks to the growing use of social media platforms such as Instagram and Twitter, plus the availability of high-tech camera mobile phones, it has become easier to create and share videos and photos. As digital video recording and uploading have become increasingly easy, digitally changed media can have significant consequences depending on the information being changed. With deepfakes, one may create hyper-realistic videos and fake pictures by using the advanced techniques from deep learning technology. Accompanying the wide use of social media, deepfakes can immediately reach millions of people and can be very dangerous to make fake news, hoaxes, and fraud [17,18]. Fake news contains bogus material that is presented in a news style to deceive people [19,20]. Bogus and misleading information spreads quickly and widely through social media channels, with the potential to affect millions of people [21]. Research shows that one in every five internet users receives news via Facebook, second only to YouTube [22]. This rise in popularity and reputation of video necessitates the initiation of proper tools to authenticate media channels and news. Considering the easy access and availability of tools to disseminate false and misleading information using social media platforms, determining the authenticity of the content is becoming difficult day by day [22]. Current issues are attributed to digital misleading information, also called disinformation, and represent information warfare where fake content is presented deliberately to alter people’s opinions [17,22,23,24].

### 1.1. Existing Surveys

Several survey and review papers have been presented on deepfake detection (Table 1). For example, ref. [25] presents a survey of deepfake creation and detection techniques. Potential trends of deepfake techniques, challenges, and future directions are discussed. The survey does not include a systematic review and contains papers for the period of 2017 to 2019 only and lacks recent papers in deepfake technology. Similarly, ref. [26] provides a survey of the deepfake approaches with respect to the type of swap used to generate deepfakes. The study discusses the papers utilizing face synthesis, identity swap, attribute manipulation, and expression swap. Another study [27] presents a systematic literature review that covers the research works related to the evolution of deepfakes in terms of face synthesis, attributes manipulation, identity swap, and expression swap. The approaches are discussed with respect to the mathematical models and the types of signals used for creating and detecting deepfakes. In addition, several current datasets are discussed that are used for testing deepfake creation and detection approaches.

Similarly, ref. [28] provides a survey of deepfake creation and detection techniques with a focus on the architecture of various networks used for this purpose. For example, detailed discussions on the capability of various deep learning networks are provided, along with their architectures used in different studies. The authors provide a survey of tools and algorithms used for deepfake creation and detection in [29]. A brief discussion on deepfake challenges, advances, and strategies is provided, however, the number of discussed studies is very small. The current study presents a systematic literature review (SLR) of deepfake creation and detection techniques and covers images and video similar to other surveys and, additionally, includes the studies related to deepfake tweets.

### 1.2. Contributions of Study

While spreading fake and misleading information is easier, its identification and correction are becoming harder [30]. For amending its impact and fighting against deepfakes, it is necessary to understand deepfakes, the technology behind them, and the potential tools and methods to identify and prevent the wide spread of deepfake videos. In this regard, this study makes the following contributions:

- A brief overview of the process involved in creating deepfake videos is provided.
- Deepfake content is discussed with respect to different categories such as video, images, and audio, as well as fake content provided in tweets. The process involved in generating these deepfakes is discussed meticulously.
- A comprehensive review of the methods presented to detect deepfakes is discussed with respect to each kind of deepfake.
- Challenges associated with deepfake detection and future research directions are outlined.

The rest of the paper is structured in three sections. Section 2 presents the survey methodology, while the process of deepfake creation is described in Section 3. Section 4 discusses the types of deepfakes and the potential methods to detect such deepfakes. Discussions and future directions are provided in Section 5 and Section 6 while the conclusions are given at the end.

## 2. Survey Methodology

**Figure 1:**
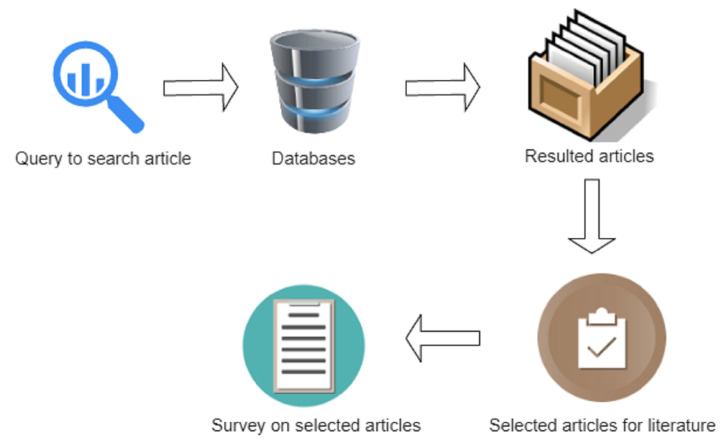
Research article search and selection methodology.

The first and foremost step in conducting a review is searching and selecting the most appropriate research papers. For this paper, both relevant and recent research papers need to be selected. In this regard, this study searches the deepfake literature from Web of Science (WoS) and Google Scholar which are prominent scientific research databases. Figure 1 shows the methodology used for research article search and selection.

The goal of this systematic review and meta-analysis is to analyze the advancement of deepfake detection techniques. The study also examines the deepfake creation methods. The study is carried out following the Preferred Reporting Items for Systematic Reviews and Meta-Analyses (PRISMA) recommendations. A systematic review helps scholars to gain a thorough understanding of a certain research area and provides future insights [31]. It is also known for its structured method for research synthesis due to its methodological process and identification metrics in identifying relevant studies when compared to conventional approaches [32]. This makes it a valuable asset not only for researchers but also for post-graduate students in developing an integrated platform for their research studies by identifying existing research gaps and the recent status of the literature [33].

### 2.1. PRISMA

PRISMA is a minimal set of elements for systematic reviews and meta-analyses that is based on evidence [34]. PRISMA consists of 4 phases and a checklist of 27 items [35,36]. PRISMA is generally designed for reporting reviews that evaluate the impact of an intervention, although it may also be utilized for a literature review with goals other than assessing approaches (e.g., evaluating etiology, prevalence, diagnosis, or prognosis). PRISMA is a tool that writers may use to enhance the way they report systematic reviews and meta-analyses [34]. It is a valuable tool for critically evaluating published systematic reviews, but it is not a quality evaluation tool for determining a systematic review’s quality.

### 2.2. Information Source

The literature search for this study includes research papers from peer-reviewed journals that are indexed in the Science Citation Index Expanded (SCIE), Social Science Citation Index (SSCI), and Arts & Humanities Citation Index (AHCI). The search query is developed after a review of the literature and refined to obtain the most relevant papers. The databases are chosen for their reliability and scientific rigor, and they are judged to be adequate and most appropriate for our evaluation.

### 2.3. Search Strategy

The search was conducted in two stages: the first in February 2021, following the conclusion of the manuscript’s primary results; and the second in May 2021, to guarantee that more updated and recent material is included. In all of the utilized databases, Boolean operators are employed for the search, and three groups of queries are used in the procedure, as follows:

Keywords to search papers related to Deepfake in different databases: *‘Deepfake’, ‘Deepfake Detection’, ‘Deepfake creation’, ‘fake videos’, ‘fake tweets’*.

### 2.4. Inclusion Criteria

The following inclusion criteria are used in the selection of the articles:

1. Studies that applied machine learning algorithms.
2. Studies that applied deep learning algorithms.
3. Studies that evaluated fake image detection, fake video detection, fake audio detection, and fake tweet detection.
4. Studies that used algorithms to analyze deepfakes using physiological and biological signals.

### 2.5. Exclusion Criteria

The following studies are excluded:

1. Studies that used any machine learning or deep learning approaches for problems that are not directly related to deepfake detection.
2. Studies that used other techniques or classic computer vision approaches and do not focus on deepfake detection.
3. Studies that did not provide a clear explanation of the machine learning or deep learning model that was used to solve their problem.
4. Review studies.

The use of the word ’fake’ resulted in many irrelevant papers such as those on fake news, fake tweets, fake articles, and fake images. Therefore, these studies are also excluded.

### 2.6. Study Selection

The initial number (*n* = 158) is obtained by an article search. Initially, 8 articles are excluded including 3 each in Russian and Spanish languages, and 1 each in Chinese and Portuguese languages. This is followed by the elimination of duplicated articles (*n* = 50). Afterward, from the resulting data, articles’ abstracts are studied to refine the selection. For this purpose, the focus is on the studies that are most relevant to deepfake detection and creation. In particular, the research articles are checked for the techniques used to detect and create deepfake data, and a total of (*n* = 30) are rejected based on the selection criteria. Seventy articles are assessed in their entirety, from which 10 are discarded due to the unavailability of the full text. Sixty papers met the inclusion criteria and are subjected to data extraction.

### 2.7. Data Extraction

Seven researchers collected and extracted data from each article in this study to examine and highlight the major points. Every included paper is properly examined for a variety of facts that had been predefined and agreed upon by all researchers. The extracted data consist of general study features such as the study title and publication year.

### 2.8. Quality Assessment

To guarantee that the findings of the selected publications can contribute to this review, a quality assessment method is developed. These criteria are created using the compiled list published by [37], which includes aspects of study design, data collection, data analysis, and conclusion. To the best of our knowledge, there is no agreement on the standard criteria for assessing research quality. As a result, we used the aforementioned recommendations because they have been used in several systematic reviews and encompass all aspects required to evaluate the quality of a research publication.

### 2.9. Quality Assessment Results

**Figure 2:**
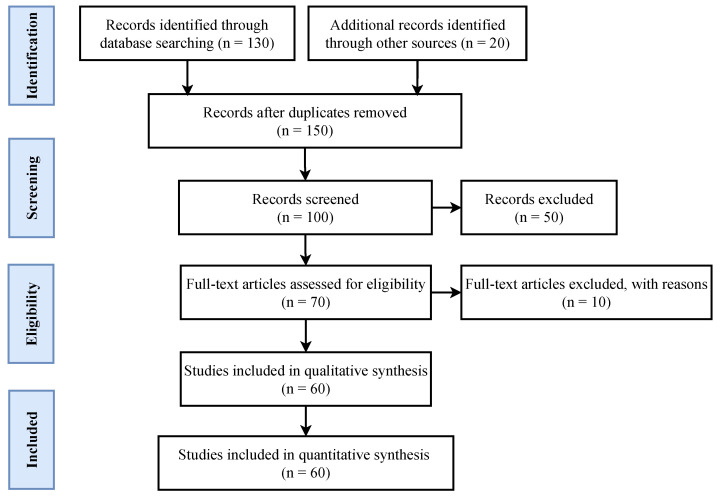
Flow chart of paper selection methodology.

Figure 2 shows the flowchart of the study design, indicating that this review has made a meaningful contribution. A breakdown of the selected research articles concerning the studied topic is provided in Table 2.

## 3. Deepfake Creation

**Figure 3:**
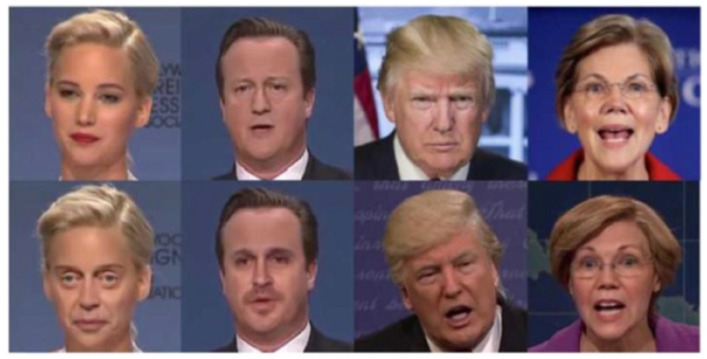
Examples of original and deepfake videos.

Figure 3 shows the original and fake faces generated by deepfake algorithms. The top row shows the original faces whereas the bottom row shows the corresponding fake faces generated by deepfake algorithms. It shows a good example of the potential of deepfake technology to generate genuine-looking pictures.

Deepfakes have grown famous due to the ease of using different applications and algorithms, especially those which are available for mobile devices. Predominantly, deep learning techniques are used in these applications, however, there are some variations as well. Data representation using deep learning is well known and has been widely used in recent times. This study discusses the use of deep learning techniques with respect to their use to generate deepfake images and videos.

### 3.1. FakeApp

**Figure 4:**
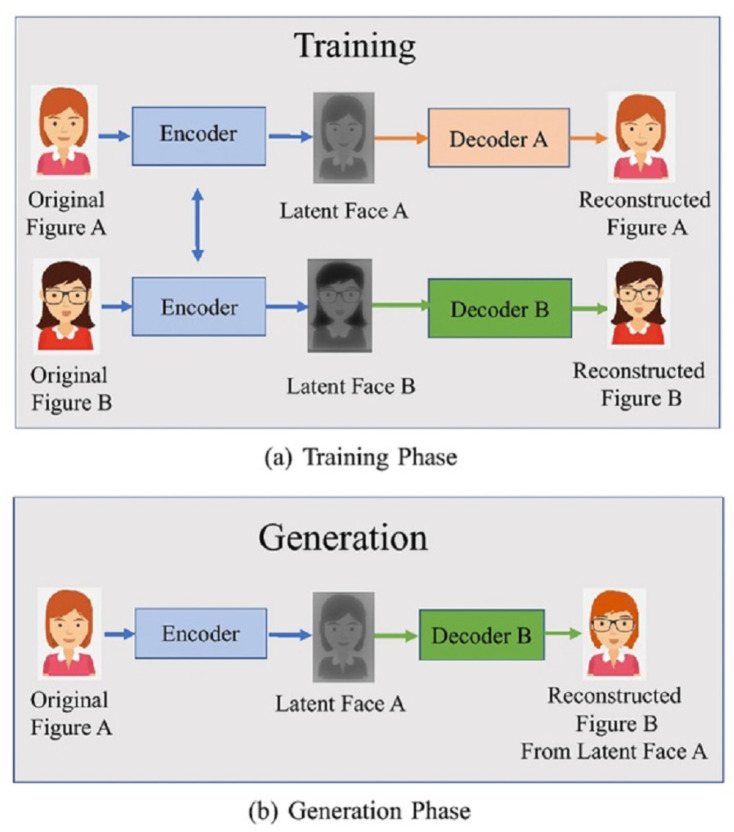
Deepfake generation process using encoder–decoder pair [40].

FakeApp is an app built by a Reddit user to generate deepfakes by utilizing an autoencoder–decoder pairing structure [38,39]. By using this technique, face pictures are decoded using an autoencoder and a decoder. Encoding and decoding pairs are required to change faces between input and output pictures. Each pair is trained on a different image collection and the encoder parameters are shared between the two networks. As a result, two pairs of encoders share the same network. Faces are often similar in terms of eye, nose, and mouth locations and, thus, this technique makes it very easy for a common encoder to learn the similarities between two sets of face pictures. Figure 4 demonstrates a deep creation process, in which the characteristics of face A are linked to decoder B to recreate face B from the original face A. It depicts the use of two pairs of encoder–decoders in a deep creational model. For the training process, two networks utilize the same encoder and various decoders (Figure 4a). The standard encoder encodes an image of face A and decoder B to generate a deepfake image (Figure 4b).

### 3.2. DeepFaceLab

**Figure 5:**
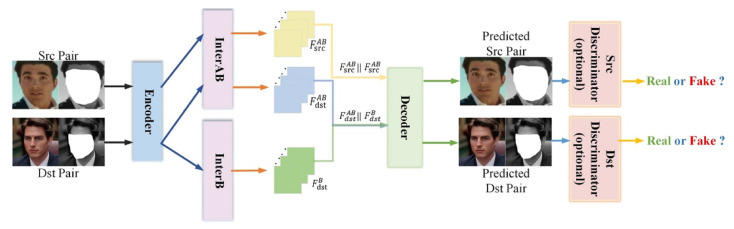
Architecture of DeepFaceLab from [41].

**Figure 6:**
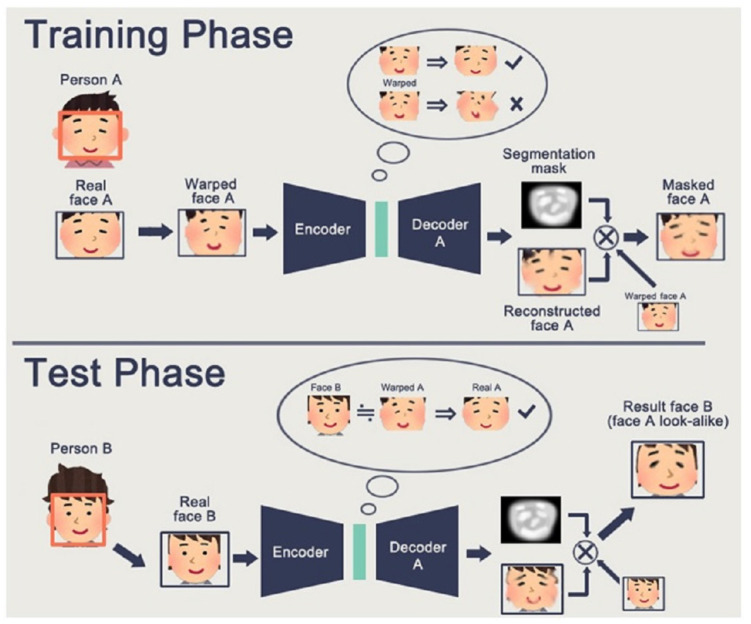
Training and testing phases of FC-GAN [46].

DeepFaceLab [41] was introduced to overcome the limitations of obscure workflow and poor performance which are commonly found in deepfake generating models. It is an enhanced framework used for face-swapping [41] as shown in Figure 5. It uses a conversion phase that consists of an ‘encoder’ and ‘destination decoder’ with an ’inter’ layer between them, and an ‘alignment’ layer at the end. The proposed approach LIAE is shown in Figure 6. For feature extraction, heat map-based facial landmark algorithm 2DFAN [42] is used while face segmentation is achieved by a fine-grained face segmentation network, TernausNet [43].

Other deepfake apps such as D-Faker [44] and Deepfake [45] use a very similar method for generating deepfakes.

### 3.3. Face Swap-GAN

Face Swap-GAN is an enhanced version of deepfakes that utilizes a GAN [5,46]. It makes use of the two kinds of losses to deep learning: adversarial loss and perceptual loss [47]. Eye movements are more natural and constant when using perceptual loss. It also helps to smooth artifacts in the segmentation masks, resulting in higher quality output videos. As a result, it is possible to create outputs with resolutions of 64 × 64, 128 × 128, and 256 × 256.

Additionally, the FaceNet implementation [48] introduces a multi-task convolutional neural network (CNN) to make face detection more reliable and face recognition more accurate. Generative networks are implemented with CycleGAN [49]. Table 3 describes some major deepfake techniques and their usual features.

### 3.4. Generative Adversarial Network

Goodfellow et al. [5] proposed GAN to analyze the underlying unknown data distribution by pushing the produced samples to be unrecognizable from actual photos. GAN is the best deepfake algorithm because it creates real images by combining two neural networks (NNs). Machine learning techniques are smart enough to learn from a collection of images and then combine these images to create an image that feels realistic to human eyes. For example, we may use GAN to produce realistic pictures of animals, clothing designs, and anything else that GAN has been trained for [50]. GAN combines two NNs, one of which is referred to as the generator and the other as the discriminator. The discriminator evaluates the images produced by the generator for authenticity, and it helps in generating more realistic images that look real to human eyes [50]. The generator tries to generate fake images from the image dataset that is provided to it, and it tries to generate realistic images [5].

### 3.5. Encoder/Decoder

The encoder is involved in the process of converting data into the desired format while the decoder refers to the process of converting coded data into an understandable format. Encoder and decoder networks have been extensively researched in machine learning [51] and deep learning [52], especially for voice recognition [53] and computer vision tasks [54,55]. A study [54] used an encoder–decoder structure for semantic segmentation. The proposed technique overcomes the constraints of prior methods based on fully convolutional networks by including deconvolutional networks and pixel-wise prediction, allowing it to identify intricate structures and handle objects of numerous sizes. An encoder is a network (FC, CNN, RNN, etc.) that receives input and produces a feature map/vector/tensor as output. These feature vectors contain the data, or features, that represent the input. The decoder is a network (typically the same network structure as the encoder, but in the opposite direction) that receives the feature vector from the encoder and returns the best close match to the actual input or intended output [56]. The decoders are used to train the encoders. There are no tags (hence unsupervised). The loss function computes the difference between the real and reconstructed input. The optimizer attempts to train both the encoder and the decoder to reduce the reconstruction loss [56].

## 4. Deepfake Detection

**Figure 7:**
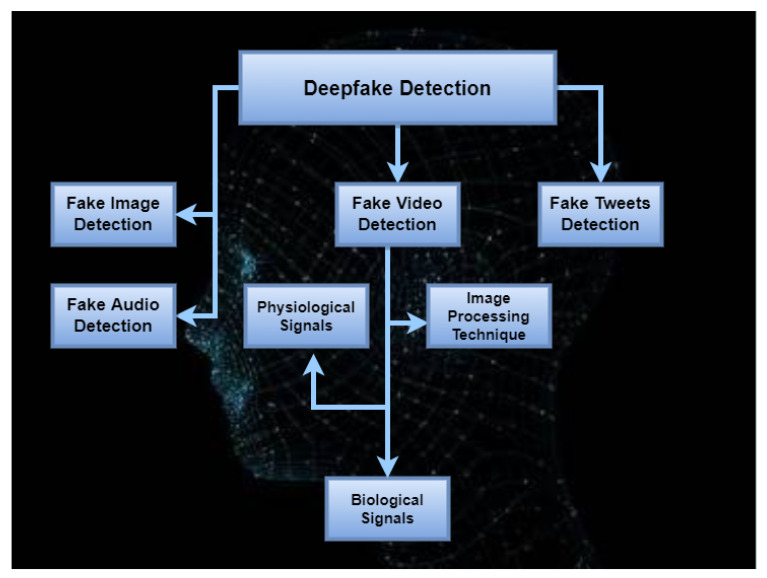
Types of deepfake videos and detection process.

Deepfakes can compromise the privacy and societal security of both individuals and governments. In addition, deepfakes are a threat to national security, and democracies are progressively being harmed. To overcome/mitigate the impact of deepfakes, different methods and approaches have been introduced that can identify deepfakes and appropriate corrective actions can be taken. This section gives a survey of deepfake detection approaches with respect to deepfake classification into two main categories (Figure 7): deepfake images and deepfake videos, with the addition of deepfake audio and deepfake tweets as well.

### 4.1. Deepfake Video Detection

A study [62] proposed LipForensics, a detection method for detecting forged face videos. LipForensics focuses on high-level semantic anomalies in mouth motions, which are prevalent in many created films. For training, the study used the FaceForensics++ (FF++) dataset, which includes 1.8 million modified frames and 4000 false videos created by two face-swapping algorithms, DeepFakes (DF) and FaceSwap (FS), as well as two face reenactment methods, Face2Face and NeuralTextures (NT). DeeperForensics (DFo) and FaceShifter (FSh), face-swapping, Celeb-DF-v2 (CDF), and DeepFake Detection Challenge (DFDC) datasets are used as a testing dataset. The study obtained 82.4%, 73.5%, 97.1%, and 97.6% accuracy from CDF, DFDC, FSh, and DFo, respectively.

A study [63] proposed deepfake detection as a fine-grained classification issue and presented a novel multi-attentional deepfake detection network. The study is composed of three major components. First, numerous spatial attention heads to direct the network’s attention to distinct local portions, second, textural feature enhancement block to zoom in on subtle artifacts in shallow features, and, third, combining low-level textural and high-level semantic characteristics driven by attention maps. The study used FF++, DFDC, and CDF datasets, where FF++ achieved 97.60% accuracy, CDF achieved 67.44% accuracy, and DFDC achieved 0.1679 Logloss.

Along the same lines, [64] proposes Multi-Feature Fusion Network (MFF-Net), a deepfake detection system that combines RGB features, and textural information extracted by an NN and signal processing techniques. The proposed system is composed of four major modules: (1) a feature extraction module to extract textural and frequency information, (2) a texture augmentation module to zoom the subtle textural features in shallow layers, (3) an attention module, and (4) two instances of feature fusion. Feature fusion includes fusing textural features from the shallow RGB branch and feature extraction module and fusing the textural features and semantic information. For the experimental process, the study used DFD, CDF, and FF++ datasets where FF++ achieved 99.73% accuracy, DFD achieved 92.53% accuracy, and CDF achieved 75.07% accuracy.

The authors propose Fake-Buster in [65], to address the issue of detecting face modification in video sequences using recent facial manipulation techniques. The study used NN compression techniques such as pruning and knowledge distillation to construct a lightweight system capable of swiftly processing video streams. The proposed technique employs two networks: a face recognition network and a manipulation recognition network. The study used the DFDC dataset which contains 119,154 training videos, 4000 validation videos, and 5000 testing videos. The study achieved 93.9% accuracy. Another study [66] proposed media forensics for deepfake detection using hand-crafted features. To build deepfake detection, the study explores three sets of hand-crafted features and three distinct fusion algorithms. These features examine the blinking behavior, the texture of the mouth region, and the degree of texture in the picture foreground. The study used TIMIT-DF, DFD, and CDF datasets. The evaluation results are obtained by using five fusion operators: concatenation of all features (feature-level fusion), simple majority voting (decision-level fusion), decision-level fusion weighted based on accuracy using TIMIT-DF for training, decision-level fusion weighted based on accuracy using DFD for training, and decision-level fusion weighted based on accuracy using CDF for training. The study concludes that hand-crafted features achieved 96% accuracy.

A lightweight 3D CNN is proposed in [67]. This framework involves the use of 3D CNNs for their outstanding learning capacity in integrating spatial features in the time dimension and employs a channel transformation (CT) module to reduce the number of parameters while learning deeper levels of the extracted features. To boost the speed of the spatial–temporal module, spatial rich model (SRM) features are also used to disclose the textures of the frames. For experiments, the study used FF++, DeepFake-TIMIT, DeepFake Detection Challenge Preview (DFDC-pre), and CDF datasets. The study achieved 99.83%, 99.28%, 99.60%, 93.98%, and 98.07% accuracy scores using FF++, TIMIT HQ, TIMIT LQ, DFDC-pre, and CDF datasets, respectively.

#### 4.1.1. Deepfake Video Detection Using Image Processing Techniques

Several approaches have been presented to detect deepfake videos based on facial landmarks, e.g., Dlib [68] detector and multi-task convolutional neural network (CNN) [69,70], etc. For example, a deep learning model has been proposed by [71] to detect deepfake videos. The study uses the FaceForensics dataset to train a CNN. The FaceForensics++ dataset comprises real and fake videos, of which 363 videos are real videos and 3068 are fake videos. Additionally, the model is tested with a variety of AI approaches including layer-wise relevance propagation (LRP) and local interpretable model-agnostic explanations (LIMEs) to provide clear representations of the prominent parts of the picture as pointed out by the model. Firstly, the face is extracted from the images using Dlib. After that, CNN XceptionNet is used to extract the features. To analyze the results of the models, the study utilizes the Xception network which is a conventional CNN with depth-wise separable convolutions (DWSCs) and LIME. Using 1.3× and 2× background scales, the study trains the algorithm with 90.17% accuracy on the image dataset. With the experimental process at 1.3×, the model achieves 94.33% accuracy, and at 2× the model achieves 90.17% accuracy.

A study [72] proposes a system for detecting deepfake videos using a support vector machine (SVM). For detecting fake videos, the model utilizes feature points (FPs) taken from the video to build an AI model. Different FP extraction methods have been used for experiments including histogram of oriented gradient (HOG), oriented features from accelerated segment test (FAST), rotated binary robust independent elementary features (BRIEF), oriented robust binary (ORB), binary robust invariant scalable key-points (BRISK), KAZE, speeded-up robust features (SURF). The study uses the dataset from [73] which comprises 90 MP4 videos with a length of about 30 s. Half of the videos in the collection are fake while the other half are real. The HOG FP extraction method obtains a 95% accuracy whereas ORB achieves 91% accuracy, SURF achieves 90.5% accuracy, BRISK 87%, FAST 86.5%, and KAZE 76.5% accuracy. The results show that the HOG extracted FP shows the highest accuracy.

Xin et al. [74] propose a deepfake detection system based on inconsistencies found in head poses. The study focuses on how deepfakes are created by splicing a synthetic face area into the original picture, as well as how it can employ 3D posture estimation for identifying manufactured movies. The study points out that the algorithms are employed to generate various people’s faces but without altering their original expressions. This leads to mismatched facial landmarks and facial features. As a result of this deepfake method, the landmark positions of a few fake faces may sometimes differ from those of the actual ones. They may be distinguished from one other based on the difference in the distribution of the cosine distance between their two head orientation vectors. The study uses Dlib to recognize faces and retrieve 68 facial landmarks. OpenFace2 is used to build a standard face 3D model, from which a difference calculation is made based on that model. The suggested system makes use of the UADFV data. In this case, an SVM classifier using radian basis function (RBF) kernels is utilized. When using an SVM to classify the UADFV dataset, the SVM classifier achieved an area under the receiver operating characteristic curve (AUROC) of 0.89.

**Figure 8:**
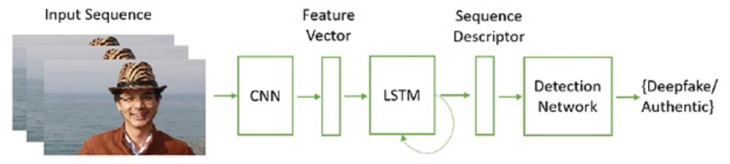
Deepfake detection using CNN and LSTM [75].

An automated deepfake video detection pipeline based on temporal awareness is proposed in [75], as shown in Figure 8. For this purpose, the study proposes a two-stage analysis where the first stage involves using a CNN to extract characteristics at the frame level, followed by a recurrent neural network (RNN) that can detect temporal irregularities produced during the face-swapping procedure. The study created a dataset of 600 videos, half of which are gathered from a variety of video-hosting websites, while the other 300 are random selections from the HOHA dataset. With the use of sub-sequences of n=20,40,80 frames, the performance of the proposed model is evaluated in terms of detection accuracy. The results show that from the selected frames, CNN-LSTM achieves the highest accuracy of 97.1% from 40 and 80 frames.

Several patterns and clues may be used to investigate spatial and temporal information in deepfake videos. For example, a study [76] created FSSPOTTER to detect swapped faces in videos. The spatial feature extractor (SFE) is used to distribute the videos into multiple segments, each of which comprises a specified number of frames. Input clips are sent into the SFE, which creates frame-level features based on the clips’ frames. Visual geometry group (VGG16) convolution layers with batch normalization are used as the backbone network to extract spatial information in the intra-frames of the image. The superpixel-wise binary classification unit (SPBCU) is also used with the backbone network to retrieve additional features. An LSTM is used by the temporal feature aggregator (TFG) to detect temporal anomalies inside the frame. In the study, the probabilities are computed by using a fully connected layer, as well as a softmax layer to determine if the clip is real or false. For the evaluation process, the study uses FaceForensics++, Deepfake TIMIT, UADVF, and Celeb-DF datasets. FSSPOTTER achieves a 91.1% accuracy from UADFV, Celeb-DF 77.6%, Deepfake TIMITHQ 98.5%, and LQ 99.5%, whereas using FaceForensics++, FSSPOTTER obtains 100% accuracy. In [77], the CNN-LSTM combo is used to identify and classify the videos into fake and real. Deepfake detection (DFD), Celeb-DF, and deepfake detection challenge (DFDC) are the datasets utilized in the analysis. The experimental process is performed with and without the usage of transfer learning. The XceptionNet CNN is used for detection. The study combined all three datasets to make predictions. Using the proposed model on the combined dataset, an accuracy of 79.62% is achieved without transfer learning, and with transfer learning the accuracy is 86.49%.

A YOLO-CNN-XGBoost model is presented in [10] for deepfake detection. It incorporates a CNN, extreme gradient boosting (XGB), and the face detector you only look once (YOLO). As the YOLO face detector extracts faces from video frames, the study uses the InceptionResNetV2 CNN to extract facial features from the extracted faces. The study uses the CelebDF-FaceForencics++ (c23) dataset which is a combination of two popular datasets: Celeb-DF and FaceForencics++ (c23). Accuracy, specificity, precision, recall, sensitivity, and F1 score are used as evaluation parameters. Results indicate that the CelebDF-FaceForencics++ (c23) combined dataset achieves a 90.62% area under the curve (AUC), 90.73% accuracy, 93.53% specificity, 85.39% sensitivity, 85.39% recall, and 87.36% precision. The model obtains an average F1 score of 86.36% for the combined dataset.

#### 4.1.2. Deepfake Video Detection Using Physiological Signals

Besides using the image processing approaches for detecting deepfake videos, physiological signals can also be used for the same purpose. For example, a study [78] proposed a system to identify deepfake videos using mouth movements. For this purpose, the study used a CNN and designed a deepfake detection model with mouth features (DFT-MF). To detect deepfakes, two different datasets are used, the deepfake forensics dataset and the VID-TIMIT dataset, that contain real and fake videos. The deepfake forensic dataset comprises a total of 1203 videos with 408 real videos and 795 fake videos, whereas the VID-TIMID dataset comprises 320 low-quality (LQ) and 320 high-quality (HQ) videos. In the preprocessing step, a Dlib classifier is utilized to recognize face landmarks. As an example, the face according to Dlib has (49, 68) coordinates. A person’s eyebrows, nose, and other facial features can be determined using the Dlib library. Afterward, all frames with a closed mouth are excluded by measuring the space between lips. According to the suggested model, sentence length is determined by the number of words that are spoken. The study also focuses on the rate of speech and shows that about 120–150 words per minute are spoken. In the proposed system, deepfake videos are determined by facial emotions and speech speed. The experimental results of DFT-MF show that, using the deepfake forensics dataset, a 71.25% accuracy can be obtained, whereas with the deepfake Vid-TIMIT dataset, DFT-MF achieves a 98.7% accuracy from LQ and 73.1% accuracy from HQ.

A study [79] leverages a deep neural network (DNN) model to study fake videos and formulate a novel approach for exposing fraudulent face videos. An eye blinking signal is detected in the videos, which is a physiological signal that does not show up well in synthetically created false videos. CNN and eye aspect ratio methods are employed in the long-term recurrent convolutional neural network (LRCN) model. For this purpose, an eye blinking video dataset of 50 videos of a 30-second duration was generated. The study utilized 40 movies for training the LRCN model and 10 for testing. Results show that regarding area under reciprocal control (AUC), the result shows that LRCN performs best with 99% accuracy whereas 98% and 79% accuracy is achieved by the CNN and eye aspect ratio (EAR), respectively.

‘DeepVision’, a novel algorithm to discriminate between real and fake videos, is presented in [80] and utilizes eye blink patterns for deepfake video detection. Fast-hyperFace and EAR are used to recognize the face and calculate the eye aspect ratio. The study created a dataset based on eye blinking patterns for experiments. The features of eye blink count and eye blink duration are retrieved to determine if a video is real or a deepfake. Experimental results using DeepVision show that an accuracy of 87.5% can be obtained.

Korshunov and Marcel [1] studied baseline methods based on the discrepancies between mouth movements and voice, as well as many versions of image-based systems frequently employed in biometrics to identify deepfakes. The study found that auditory and visible features can be used for mouth movement profiles in deepfakes. An RNN based on LSTM is utilized to recognize real and fake videos and principal component analysis (PCA) and latent Dirichlet allocation (LDA) have been utilized to decrease the dimensions of the blocks of data. In the second case, i.e., voice movements, the study used two detection methods, raw faces as features and image quality measurements (IQMs). For this purpose, 129 features were investigated including signal-to-noise ratio, specularity, blurriness, etc. The final categorization was based on PCA-LDA or SVM. For the deepfake TIMIT database, the study suggested that the detection techniques based on IQM+SVM produced the best results of 3.3% low-quality energy efficiency ratio (LQ EER) and 8.9% high-quality EER (HQ EER).

#### 4.1.3. Deepfake Video Detection Using Biological Signals

Biological signals have been predominantly used in the medical field to determine the physical and emotional state of people [81]. Using the features from the data indicating heart rate, galvanic skin response, electrocardiogram, etc., abnormal biological signals can be identified by experts. For medical analysis, such approaches require the use of sensors and nodes which are placed on different limbs of the human body; this is not possible for deepfake detection. Intuitively, computer experts have designed algorithms that can measure biological signals using features from the video data such as changes in color, motion, subtle head movements, etc. [82].

Besides the physiological signals gathered from the videos, biological signals present a potential opportunity to identify deepfakes. For example, a study [83] detected deepfakes from videos using a ‘FakeCatcher’ system. The study proposed a FakeCatcher technique for detecting synthesized information of portrait videos as a deep fake prevention solution. This method is based on the findings that biological signals collected from face regions are poorly retained geographically and temporally in synthetic content. Different methods are proposed for enhancements to the derived PPG signal’s quality, as well as the extraction process’s reliability. Chrominance attributes [84], green channel elements [85], optical properties [86], Kalman filters [87], and distinct face regions [85,86,88] are some of the suggested enhancements. The study used six biological signals, GL, GR, GM, CL, CR, and CM, where GL represents green left cheek, GR represents green right cheek, GM is the green mid-region, CL represents chrominance left, CR represents chrominance right, and CM represents chrominance mid-region. Experiments were performed using three benchmark datasets, FaceForensics, FaceForensics++, and CelebDF, in addition to the newly collected dataset, Deep Fakes (DF). The DF dataset comprises 142 portrait videos collected ’in the wild’ where each video has a length of 32 min. For detection, the study uses a CNN classifier trained on the above-mentioned features. Results indicate that the CNN achieves a 91.07% accuracy from the DF dataset, 96% accuracy for the FaceForensics dataset, 91.59% from the CelebDF dataset, and 94.65% using the FaceForensics++ dataset.

The authors of [89] discussed several biological signals for deepfake video detection, eye and gaze properties, by which deepfakes differ. Furthermore, the researchers combined those characteristics into signatures and compared original and fake videos, generating geometric, visual, metric, temporal, and spectral variances. The study used FaceForensics++, Deep Fakes, CelebDF, and DeeperForensics datasets. To categorize any video in the wild as false or real, the researchers used a deep neural network. With the proposed approach, 80.0% accuracy on FaceForensics++, 88.35% with Deep Fakes (in the wild), 99.27% using CelebDF, and 92.48% using the DeeperForensics dataset can be obtained.

The research described in [90] offers a method for not only distinguishing deepfakes from the original movies but also presents a generative model that underpins a deepfake. DL techniques are used to categorize deepfakes using CNNs. The authors found that such manipulative artifacts from biological signals can be used to detect deepfakes. The findings reveal that spatial–temporal patterns in biological signals may be thought of as a representative projection of residuals. The results show that the method correctly detects bogus films with a 97.29% accuracy and correctly detects the source model with 93.39% accuracy.

### 4.2. Deepfake Image Detection

**Figure 9:**
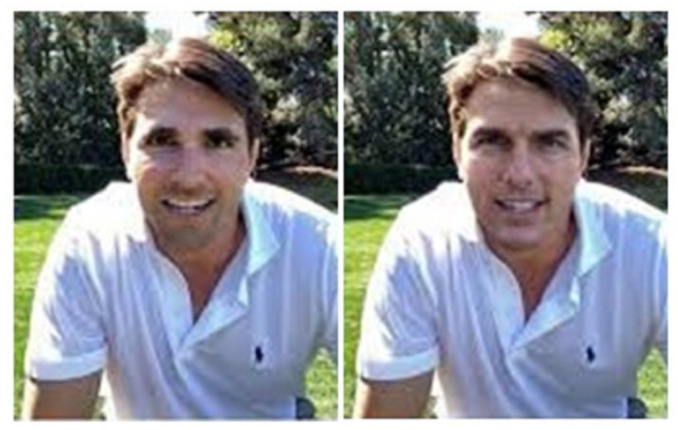
Deepfake and original image: Original image (**left**), deepfake (**right**) [92].

Unlike the detection of video deepfakes, which are sequences of images, deepfake image detection focuses on identifying a single image as a deepfake, as shown in Figure 9. For example, the study described in [91] proposed a system to detect deepfake human faces. The expectation-maximization (EM) method was used to extract features from the image. For the classification, k-nearest neighbors (k-NN), SVM, and LDA algorithms were applied. In the study, deepfake images were generated using the based approach. Fake pictures were created by five different GAN techniques, AttGAN, StarGAN, GDWCT, StyleGAN, and StyleGAN2, with the CelebA dataset as ground truth for non-fakes. For experiments, 6005 images from AttGAN, 3369 images from GDWCT, 9999 images from StyleGAN, 5648 images from StarGAN, and 3000 images from StyleGAN2 were used. The study achieved the best accuracy of 99.81% on StyleGAN2-generated images with linear SVM.

**Figure 10:**
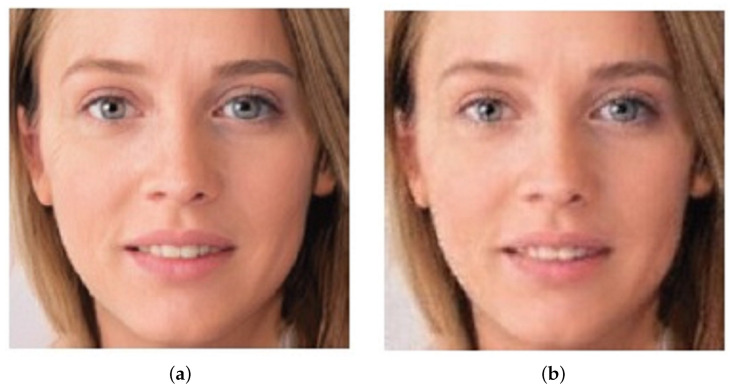
Deepfake and GANprintR-processed deepfake: (**a**) Deepfake, (**b**) deepfake after GANprintR [93].

A comprehensive evaluation of face manipulation techniques was conducted in [93] using a variety of modern detection technologies and experimental settings, including both controlled and uncontrolled scenarios. The study used four distinct deepfake image databases using different GAN variants. The StyleGAN architecture was used to create 150,000 fake faces collected online. Similarly, the 100K-faces public database that contains 80,000 synthetic faces was used. The GANprintR approach was used to remove GAN fingerprint information from the iFakeFaceDB database, which is an enhanced version of previous fake databases, as shown in Figure 10. Findings of the study reveal that an EER of 0.02% is obtained in controlled situations which is similar to the best recent research. From the iFakeFaceDB dataset, the study achieved 4.5% EER for the best fake detectors.

A method for detecting fake images was developed by Dang et al. [94]. An attention process was used to enhance the functionality of feature maps for the CNN model. Fake pictures were produced using the FaceApp software, which has up to 28 distinct filters including age, color, glasses, hair, etc. Similarly, the StarGAN method was used which has up to 40 different filters [95]. The CNN model was also tested on the study’s own collected DFFD dataset with 18,416 real and 79,960 fake pictures produced using FaceApp and StarGAN. The results were outstanding, with an EER of less than 1.0% and 99.9 percent AUC.

Along the same direction, Wang et al. [96] used the publicly accessible commercial software Face-Aware Liquify tool from Adobe Photoshop to create new faces. Additionally, skilled artists used 50 real images to produce modified images. Participants were shown fake and real photos and asked to categorize the images into groups, as part of Amazon Mechanical Turk (AMT) research. Humans were able to attain only 53.5% accuracy, which is close to chance (50 percent). Two alternative automated methods were presented against the human study: One using dilated residual networks (DRNs) to estimate whether or not the face has been distorted, and another using the optical flow field to detect where manipulation has occurred and reverse it. Using the automatic face synthesis manipulation, the study achieved 99.8% accuracy, and using the manual face synthesis manipulation, the study achieved 97.4% accuracy.

A study [97] used CNN models to detect fake face images. Different CNN techniques were used for this purpose, such as VGG16, VGG19, ResNet, and XceptionNet. For this purpose, the study used two datasets for manipulated and original images. For real images, the study used the CelebA database whereas for the fake pictures two different options were utilized. First, machine learning techniques based on GAN were used in ProGAN, and secondly, manual approaches were leveraged using Adobe Photoshop CS6 based on different features such as cosmetics, glasses, sunglasses, hair, and headwear alterations, among other things. For experiments, a range of picture sizes (from 32 × 32 to 256 × 256 pixels) were tested. A 99.99% accuracy was achieved within the machine-created scenario, whereas 74.9% accuracy was achieved from the CNN model. Another study [98] suggested detection methods for fake images using visual features such as eye color and missing details for eyes, dental regions, and reflections. Machine learning algorithms, logistic regression (LR) model and multi-layer perceptron (MLP), were used to detect the fake faces. The proposed technique was evaluated on a private FaceForensics database where LR achieved an 86.6% accuracy and the MLP achieved an 82.3% accuracy.

A restricted Boltzmann machine (RBM) is used in [99] to develop deepfake images made using facial image digital retouching. By learning discriminative characteristics, each image was classified as original or fake. The authors generated two datasets for fake images using the actual ND-IIITD Retouching (ND-IIITDR) dataset (collection B) and Celebrity Retouching (CR) which is a set of celebrity facial pictures retrieved from the internet. Fake pictures were created with the help of Max25’s PortraitPro Studio software, which took into account elements of the face such as the texture of the skin and skin tone, as well as eye coloration. In the CR and ND-IIITD Retouching datasets, the study achieved an accuracy of 96.2% and 87.1% percent, respectively.

A study [100] proposed a face X-ray, a novel image representation for identifying fraudulent face images or deepfakes. The basic finding for face X-rays is that most current face alteration algorithms share a similar step of blending a changed face into an existing backdrop picture, and there are inherent image disparities across the blending boundaries. The study used FF++ and Blending Images (BI) datasets for training and DFD, DFDC, and CDF datasets for testing. For the experimental process, the study used a CNN and achieved 95.40% accuracy from DFD, 80.92% accuracy from DFDC, and 80.58% accuracy from CDF.

### 4.3. Deepfake Audio Detection

#### 4.3.1. Fake Audio Datasets

The Fake or Real (FoR) dataset [101], which includes eight synthetically created English-accented voices using the Deep Voice 3 and Google-Wav Net generation models, was released in 2019. It is publicly available; its most important feature is that it contains sounds in two different formats, MP3 and WAV. The complete dataset consists of 198,000 files, comprising 111,000 original samples and 87,000 counterfeit samples, each lasting two seconds. The Arabic Diversified Audio [102] dataset (Ar-DAD), which was acquired via the Holy Quran audio site, was apparently a fake audio collection of Arabic speakers. The audio is of 30 male Arabian reciters and 12 mimics, and it comprises the original and mimicked voices of Quran reciters. The reciters are Arabians from Egypt, Sudan, Saudi Arabia, Yemen, Kuwait, and the United Arab Emirates. There are 379 false and 15,810 actual samples in the data, each voice has a 10 s duration.

The H-Voice dataset [103] was recently established using fake and real voices in various languages including French, English, Portuguese, Spanish, and Tagalog. It includes samples stored in the PNG format as a histogram. There are 6672 samples in this dataset and it is organized into six folders: ‘training original’, ‘training fake’, ‘validation original’, ‘validation fake’, ‘external test 1’, and ‘external test 2’. Each category has a different number of samples, where the first category has 2020 histograms while the second category contains 2088 histograms, 2016 imitations, and 72 deep voices. The third category contains 864 histograms, ‘validation fake’ contains 864 histograms, and ‘external test 1’ and ‘external test 2’ are further divided into two sub-folders, ‘fake’ and ‘original’. The ‘external test 1’ set contains a total of 760 histograms (380 fake imitation histograms and 380 original histograms) while the ‘external test 2’ set contains 76 histograms (72 fake deep voice histograms and four original histograms).

Finally, the ASV spoof 2021 challenge dataset includes two false circumstances, one cognitive and one actual. False audio is created in the cognitive environment utilizing synthetic software, whereas fake audio is created in the actual environment by replicating prerecorded sounds using sections of genuine speaker data. This dataset has not yet been released; prior versions are freely available (2015 [104], 2017 [105], and 2019 [106]).

#### 4.3.2. Deepfake Audio Detection Techniques

A large variety of methods and techniques for creating fake audio have prompted a wide interest in detecting deepfake audio in many languages. This section presents the works on recognizing mimicked and synthetically created voices. In general, there are two types of techniques that are used currently: ML and DL approaches.

Traditional ML methods are commonly used in the identification of fake audios. A study [107] created a own fake audio dataset by extracting entropy features using an imitation technique named the H-Voice dataset [103]. To distinguish between the fake and real audio, the study used ML model LR. LR achieved 98% accuracy for real vs. fake audio detection. The study points out that manual feature extraction can boost the performance of the proposed approach.

To identify artificial audio from natural human voices, Singh et al. [108] used the H-Voice dataset and suggested a quadratic SVM (QSVM) method. The study classified the audio into two types, human and AI-generated. Additional ML approaches including linear discriminant (LD), quadratic LDSVM, weighted KNN, boosted tree ensemble, and LR were compared against this model. It is observed that QSVM beats other traditional approaches by 97.56% accuracy and has only a 2.43% misclassification rate. Similarly, Borrelli et al. [109] created an SVM model using RF to classify artificial voices using a novel audio component known as short-term long-term (STLT). The Automatic Speaker Verification (ASV) spoof 2019 challenge dataset was used to train the models. The results show that RF performs best compared to SVM with a 71% accuracy result. In a similar way, [110] also used the H-Voice dataset and compared the effectiveness of SVM with the DL technique CNN to distinguish fake audio from actual stereo audio. The study discovered that the CNN is more resilient than the SVM, even though both obtained a high classification accuracy of 99%. The SVM, however, suffers from the same feature extraction issues as the LR model did.

A study [111] designed a CNN method in which the audio was converted to scatter plot pictures of surrounding samples before being input into the CNN model. The generated method was evaluated using the Fake or Real (FoR) dataset and achieved a prediction accuracy of 88.9%. Whereas the suggested model solved the generalization problem of DL-based architectures by training with various data generation techniques, it did not perform well as compared to other models in the literature. The accuracy and equal error rate (EER) were 88% and 11%, respectively, which are lower than other DL models.

Another similar study is [112] that presented deep sonar and a DNN model. The study presented the neuron behaviors of speaker recognition (SR) systems in the face of AI-produced fake sounds. In the classification challenge, their model is based on layer-wise neuron activities. For this purpose, the study used the voices of English speakers from the FoR dataset. Experimental results show that 98.1% accuracy can be achieved using the proposed approach. The efficiency of the CNN and BiLSTM was compared with ML models in [113]. The proposed approach detects imitation-based fakeness from the Ar-DAD of Quranic audio samples. The study tested the CNN and BiLSTM to identify fake and real voices. SVM, SVM-linear, radial basis function (SVMRBF), LR, DT, RF, and XGBoost were also investigated as ML algorithms. The research concludes that the SVM has a maximum accuracy of 99%, while DT has the lowest accuracy of 73.33%. Furthermore, the CNN achieves a 94.33% detection rate which is higher than BiLSTM.

### 4.4. Deepfake Tweet Detection

Similar to deepfake videos and images that are posted online as separate units, tweets posted on Twitter may also be fake, so they are also called deepfakes. Therefore, a specialized study [114] focused on detecting deepfakes from tweets alone. The study collected a dataset of deepfake tweets named the TweepFake dataset. The study collected 25,572 randomly selected tweets from 17 human accounts imitated by 23 bots. Markov chains, RNN, RNN+Markov, and LSTM are some of the approaches used to create the bots. The study used 13 deepfake detection methods: LR_BOW, RR _BOW, SVC_BOW, LR_BERT, RF_BERT, SVC_BERT, CHAR_CNN, CHAR_GRU, CHAR_CNNGRU, BERT_FT, DISTILBERT_FT, ROBERTA_FT, and XLNET_FT. Experimental results show that ROBERTA_FT performs best with an 89.6% accuracy whereas LR_BOW achieved 80.4%, RF_BOW achieved 77.2%, SVC_BOW achieved 81.1%, LR_BERT achieved 83.5%, RF_BERT achieved 82.7%, SVC_BERT achieved 84.2%, CHAR_CNN achieved 85.1%, CHAR_GRU achieved 83%, CHAR_CNNGRU achieved 83.7%, BERT_FT achieved 89.1%, DISTILBERT_FT achieved 88.7%, and XLNET_FT achieved 87.7% accuracy.

## 5. Discussion

Deepfakes have become a matter of great concern in recent times due to their large-scale impact on the public, as well as the safeguarding of countries. Often aimed at celebrities, politicians, and other important individuals, deepfakes can easily become a matter of national security. Therefore, the analysis of deepfake techniques is an important research area to devise effective countermeasures. This study performs an elaborate and comprehensive survey of the deepfake techniques and divides them into deepfake image, deepfake video, and deepfake tweet categories. In this regard, each category is separately studied and different research works are discussed with respect to the proposed approaches for detecting deepfakes. The discussed research works can be categorized into two groups with respect to the used datasets: research works using their own collected datasets for experiments and the ones making use of benchmark datasets. Table 4 contains all those research works that created their own datasets to conduct experiments for detecting deepfakes. Authors of the studies [72,74,75,79,80,83,91,93,94,96,97,99,114] created their datasets to evaluate the performance of the proposed approaches.

The authors of [72] used feature point extraction methods to create their dataset. The study created 90 MP4 videos with a length of about 30 s which were then used to perform experiments using the proposed approach for deepfake detection. Similarly, the study [74] uses facial landmarks and facial features to create a dataset containing fake images. The authors used Dlib to recognize faces and retrieve 68 facial landmarks which were used to generate fake images. To recognize deepfakes, the study used the 3D head pose method to find the dissimilarities between the genuine head pose and the head poses found in the fake images. Along the same lines, a study [79] created an eye blinking video dataset of 50 videos of 30 s duration for conducting experiments for deepfake detection. A study [75] created a dataset of 600 videos where 300 videos were deepfakes gathered from a variety of video-hosting websites while the remaining 300 were random selections from the HOHA dataset which is a publicly available deepfake dataset. From the CelebA dataset, a study [91] created a dataset by using five different GAN techniques, AttGAN, StarGAN, GDWCT, StyleGAN, and StyleGAN2. The generated dataset was later used for evaluating the performance of the proposed approach.

A study [93] used GANprintR and StyleGAN techniques to create a dataset for deepfake detection. Another study [94] used FaceApp software and the StarGAN method to create a dataset named DFFD. The generated dataset contains 18,416 real and 79,960 fake images of different celebrities. A study [96] used the Face-Aware Liquify tool provided by Adobe Photoshop to create new faces for deepfake detection by manipulating different facial features. Another study [97] used GAN, ProGAN, and Adobe Photoshop CS6 to create a dataset of fake and real images. A study [80] created a dataset by using two features, eye blink count and eye blink duration. The generated dataset was further used with several approaches to analyze the performance of deepfake detection.

By using Max25’s PortraitPro Studio software, a study [99] created two datasets, the ND-IIITD retouching (ND-IIITDR) database (collection B) and CR. Both datasets contain fake and real images of different celebrities. Real images were gathered from different online sources. Another study [83] created a DF dataset by using portrait videos. In their study, the authors used biological signals and proposed FaceChecker. Although for the same purpose of deepfake detection, another study [114] created a unique dataset for deepfake tweets where different bots are used to generate tweets about specific topics. The study collected 25,572 deepfake tweets.

Apart from the research works discussed in Table 5, several researchers have made use of the publicly available benchmark datasets for deepfakes. The authors of [1,10,71,76,77,78,98] used benchmark datasets shown in Table 5. The benchmark dataset VidTIMIT [115] consists of 35 persons speaking brief words on video and accompanying audio recordings. It can be used in experiments for automated lip-reading, multi-view face recognition, multi-modal voice recognition, and person identification, among others. The dataset was gathered in three sessions by applying a 7-day gap between the first and second sessions whereas a 6-day gap between the second and third sessions was used. The texts were picked from the TIMIT corpus’s test portion. Each individual has ten sentences. Session 1 is made up of the first six phrases (ordered alphabetically by file name); session 2 includes the following two phrases, while session 3 includes the last two.

The Celeb-DF dataset [116] has two versions, v1 and v2. The latest version, v2, comprises actual and deepfake-generated movies of comparable visual quality to those found on the internet. The Celeb-DF v2 dataset is significantly larger than the Celeb-DF v1 dataset, which only contained 795 deepfake movies. Celeb-DF now has 590 original YouTube videos with topics related to different ages, ethnic groupings, and genders, as well as 5639 videos.

DeepfakeTIMIT [117] is a collection of videos in which faces have been switched by utilizing free GAN-based software which was derived from the original autoencoder-based deepfake algorithm. The dataset was built by selecting 16 similar-looking pairings of persons from the openly available VidTIMIT database. Two alternative classifiers are trained for each of the 32 subjects: lower quality with a 64 × 64 input/output size model and higher quality with a 128 × 128 size model. As each individual had ten movies in the VidTIMIT database, 320 videos are created for each version, totaling 620 videos with faces changed.

FaceForensics++ is a forensics dataset [118] comprising 1.8 million modified frames, 1000 original videos, and 4000 false video sequences which are changed using four automated face manipulation methods: Deepfakes, Face2Face, FaceSwap, and NeuralTextures. The data came from 977 YouTube videos, all of which include a trackable mainly frontal face with no occlusions, allowing automated tampering methods to create plausible forgeries.

UADFV is another publicly available dataset [119]. This dataset is a collection of 49 actual YouTube videos that were used to make 49 false videos using the FakeApp mobile application, replacing the original face with Nicolas Cage’s visage in each of them. As a result, in all fraudulent videos, just one identity is considered. In this dataset, each video depicts a single person, with a typical resolution of 294 × 500 pixels and an average duration of 11.14 s.

The authors of [78] used two datasets for their research including the deepfake forensics dataset and the Vid-TIMIT dataset. The deepfake forensics dataset contains 1203 videos with 408 real videos and 795 deepfake videos whereas the VID-TIMID dataset comprises 320 LQ and 320 HQ videos. The study used a CNN and designed a deepfake detection model with mouth feature DFT-MF to detect deepfakes. Refs. [71,98] used the FaceForensics dataset for the detection of deepfakes. The FaceForensics dataset contains real and fake videos; 363 and 3068 videos are real and fake, respectively.

A study [71] used Dlib to extract faces from videos and CNN XceptionNet to extract features. A study [71] used a CNN to detect deepfakes whereas another study [98] used machine learning algorithms LR and MLP to detect the fake faces. Another study [1] used the deepfake TIMIT benchmark dataset. A study used PCA-LDA and SVM to extract face features and image quality measurements. The study shows that IQM+SVM performs best for fake face detection. Another study [76] used FaceForensics++, deepfake TIMIT, UADVF, and Celeb-DF benchmark datasets. FSSPOTTER is used to detect fake faces. A study [77] used deepfake detection (DFD), Celeb-DF, and deepfake detection challenge (DFDC) datasets. For the experimental process, the study used CNN XceptionNet with and without transfer learning. The Celeb-DF-FaceForensics++ (c23) dataset is used in [10] and is the combination of two datasets: Celeb-DF and FaceForensics++ (c23). For the detection of deepfakes, the study used YOLO-CNN-XGBoost.

## 6. Future Directions

Deepfakes, as the name suggests, involve the use of deep learning approaches to generate fake content, therefore, deep learning methods are desired to effectively detect deepfakes. Although the literature on deepfake detection is sparse, this study discusses several works for deepfake detection. Most of the discussed research works leverage deep learning models directly or indirectly to discriminate between real and fake content such as images and videos. Predominantly, a CNN is used either as the final classifier or feature extraction level for deepfake detection. Similarly, machine learning models SVM and LR are also utilized along with the deep learning models. Other variants of deep learning models are also investigated such as YOLO-CNN, CNN with attention mechanism, and RF and LR variants.

For most of the discussed research works, the studies utilize their collected datasets which may or may not be publicly available, so the repeatability and reproducibility of the experiments may be poor. More experiments on the publicly available benchmark datasets are needed. The knowledge of the tools used to generate deepfakes plays a vital role to determine a proper tool/model for deepfake detection, which is helpful but not very practical for real-world scenarios. For benchmark datasets, such information should not be available so that exhaustive experiments can be conducted to devise robust and efficient approaches to determine fake content.

Although changes in images can be determined using the digital signatures found in fake content, this is very difficult for deepfake content. Several indicators such as head pose, eye blink count and duration, cues found in teeth placement, and other facial landmarks are used to detect deepfakes and, with the advancement in deep learning technology, such indicators will become less prevalent in future deepfakes. Consequently, more indicators at a refined scale will be required for future deepfakes.

Keeping in view the rapid increase in and wide use of social media, the content to make deepfakes will become easier and deepfake use more widespread in the future. This means that more robust and efficient deepfake detection methods are required to work in real time, which is not in practice yet. Therefore, special focus should be placed on approaches that can work in real time. This is important as deepfake technology has shown the potential to do damage that is irreparable. Often, the damage is done before it is realized that the posted content is not real. Coupled with the speed of social media, the damage is manifold and dangerously fast. Therefore, effective, robust, and reliable approaches are needed to perform real-time deepfake detection. Due to their resource-hungry nature, deep learning approaches cannot be deployed on smartphones which are a major source of content sharing on social media. For quick and timely detection of fake content on smartphones, novel methods are needed that can be deployed on smartphones.

## 7. Conclusions

Deepfake content, both images and videos, has grown at an unprecedented speed during the past few years. The use of deep learning approaches with the wide availability of images, audio, and videos from social media platforms can create fake content that can threaten the goodwill, popularity, and security of both individuals and governments. Manipulated facial images and videos created using deepfake techniques may be quickly distributed through the internet, especially social media platforms, endangering societal stability and personal privacy. As a safeguard against such threats, commercial firms, government offices, and academic organizations are devising and implementing relevant countermeasures to alleviate the harmful effects of deepfakes.

This study aims to highlight the recent research in deepfake images and video detection, such as deepfake creation, various detection algorithms on self-made datasets, and existing benchmark datasets. This study provides a comprehensive overview of the approaches that have been presented in the literature to detect deepfakes and thus help to mitigate the impact of deepfakes. Analysis indicates that machine and deep learning models such as the CNN and its variants, SVM, LR, and RF and its variants are quite helpful to discriminate between real and fake content in the form of both images and videos. This study elaborates on the methods to counter the threats of deepfake technology and alleviate its impact. Analytical findings recommend that deepfakes can be combated through legislation and corporate policies, regulation, and individual action, training, and education. In addition to that, evolution of technology is desired for identification and authentication of the content on the internet to prevent the wide spread of deepfakes. To overcome the challenge of deepfake identification and mitigate their impact, large efforts are needed to devise novel and intuitive methods to detect deepfakes that can work in real time.

## Figures and Tables

**Table 1:** A comparative analysis of review/survey papers on deepfakes.

Reference	Type	DF Detection	DF Creation	DF Tweets	Timeline	Published	Scope
[25]	Survey	Image/Video	Image/Video	No	2019	Arxiv	Covers deepfake creation and detection approaches presented from 2017 to 2020, however, there are few studies from 2020.
[26]	Survey	Image	Image	No	2020	Elsevier	The survey covers the studies on face manipulation approaches only and does not include deepfake video creation and detection.
[27]	Survey	Image/Video	Image/Video	No	2021	Arxiv	Recent studies on face synthesis, attribute manipulation, identity swap, and expression swap are discussed, in addition to the deepfake datasets.
[28]	Survey	Image/Video	Image/Video	No	2021	ACM	Focuses on the potential of various deep learning networks for creating and detecting deepfakes. Similarly, well-known architectures from different studies are discussed.
[29]	Survey	Image/Video	Image/Video	No	2021	Springer	Covers a brief overview of different deepfake creation and detection tools and covers a small range of studies.
Current	SLR	Image/Video	Image/Video	Yes	2021	Sensors	Focuses on the recent works regarding deepfake creation and detection techniques. In addition to images and videos, it covers deepfake tweets. Many recent studies are covered regarding famous deepfake apps and approaches.

**Table 2:** Details of the selected articles with respect to sub-topics.

Topic	No. of Articles
Deepfake Creation	18
Deepfake Detection	20
Deepfake Video Detection Using Image Processing Techniques	7
Deepfake Video Detection Using Physiological Signals	4
Deepfake Video Detection Using Biological Signals	1
Deepfake Audio Detection	5
Deepfake Image Detection	7
Deepfake Tweet Detection	1
Total	58

**Table 3:** Brief overview of deepfake face apps.

Tool	Link & Key Features
DeepFaceLab	–https://github.com/iperov/DeepFaceLab. –Reduced training time of 3 h. –Better performance for pose and expression adaptation. –Sharp facial landmarks such as eyes and teeth. –Supports large scale dataset of up to 100 k images to improve image quality. –Supports lip manipulation, head replacement and do-age, etc.
FSGAN	–https://github.com/YuvalNirkin/fsgan. –Face-swapping is unified with reenactment model that may be used for any pair of faces without the need for training on the pair of faces in question – Adapt to changes in both position and emotion [57].
DiscoFaceGAN	–https://github.com/microsoft/DiscoFaceGAN. –Generates face pictures of virtual individuals with latent characteristics such as identity, expression, posture, and lighting that are independent of each other. –Consider using 3D priors in adversarial learning [58].
FaceShifter	–https://lingzhili.com/FaceShifterPage. –Facial swapping in high fidelity by leveraging and combining the target features. Any fresh face combination can be used without specialized training [59].
AvatarMe	–https://github.com/lattas/AvatarMe. –From random ‘in-the-wild’ photos, creates a 3D face. A single low-quality picture may be used to rebuild a 3D face with a resolution of 4 K or 6 K. [60]
“Do as I Do” Motion Transfer	–github.com/carolineec/EverybodyDanceNow. –By learning a video-to-video translation, one can automatically transmit motion from one person to another. –Can generate a dance movie with numerous people that is motion-synchronized [61].

**Table 4:** Comparison table of self-made datasets.

Reference	Dataset	Classifier	Method	Performance
[72]	Own dataset created [73]	SVM	FP extraction method: HOG, ORB, SURF, BRISK, FAST, KAZE	HOG 95%, ORB 91%, SURF 90.5%, BRISK 87%, FAST 86.5%, & KAZE 76.5%
[74]	UADFV	SVM	3D head pose	97.4% AUC
[79]	Self-made dataset	DNN	Eyeblink + LRCN	99% AUC
[75]	Own dataset	CNN and LSTM	CNN_LSTM	97.1% AUC
[91]	Self-made dataset	KNN, SVM, and LDA	AttGAN, StarGAN, GDWCT, StyleGAN and StyleGAN2	99.81% from StyleGAN2 with SVM
[93]	100K-Faces (StyleGAN) and iFakeFace DB	Deep learning	CNN	EER = 0.3% from 100K-Faces (StyleGAN), EER = 4.5% from iFakeFace DB
[94]	DFFD (ProGAN, StyleGAN)	Deep learning	CNN + attention mechanism	AUC = 100%, EER = 0.1%
[96]	Own (Adobe Photoshop)	Deep learning features	DRN	AP = 99.8%
[97]	Own (ProGAN, Adobe Photoshop)	Deep learning features	CNN	AUC = 99.9%, AUC = 74.9%
[80]	Self-made dataset	Machine learning	Eye blinking	87.5%
[99]	Own (Celebrity Retouching, ND-IIITD Retouching)	Deep learning features (face patches)	RBM	CR= 96.2%, ND-IIITDR = 87.1%
[114]	Own TweepFake	Machine learning	LR_BOW, RF_BOW, SVC_BOW, LR_BERT, RF_BERT, SVC_BERT, CHAR_CNN, CHAR_GRU, CHAR_CNNGRU, BERT_FT, DISTILBERT_FT, ROBERTA_FT, and XLNET_FT	ROBERTA_FT 89.6%, LR_BOW 80.4%, RF_BOW 77.2%, SVC_BOW 81.1%, LR_BERT 83.5%, RF_BERT 82.7%, SVC_BERT 84.2%, CHAR_CNN 85.1%, CHAR_GRU 83%, CHAR_CNNGRU 83.7%, BERT_FT 89.1%, DISTILBERT_FT 88.7%, and XLNET_FT 87.7%
[83]	Own Deep Fakes dataset	CNN	Biological signals	91.7%

**Table 5:** Comparison of benchmark datasets.

Reference	Dataset	Classifier	Method	Performance
[78]	Deepfake Forensics Vid-TIMIT dataset	CNN	DFT-MF	Deepfake Forensics dataset 71.25% Vid-TIMIT dataset LQ 98.7% & HQ 73.1%
[71]	FaceForensics++ dataset	CNN	CNN XceptionNet	At 1.3x background scale 94.33% accuracy At 2x background scale 90.17% accuracy
[1]	DeepfakeTIMIT (LQ) DeepfakeTIMIT (HQ)	PCA+RNN PCA+LDA SVM	Audio-visual features	DeepfakeTIMIT (LQ) EER = 3.3% DeepfakeTIMIT (HQ) EER = 8.9%
[98]	FaceForensics dataset	Logistic regression MLP	Visual features	86.6% LR 82.3% MLP
[76]	FaceForensics++, DeepfakeTIMIT, UADVF, and Celeb-DF datasets	CNN, LSTM	FSSPOTTER	FaceForensics++ 100%, DeepfakeTIMIT (LQ) 99.5% DeepfakeTIMIT (HQ) 98.5%, UADVF 91.1% and Celeb-DF 77.6%
[77]	DFD Celeb-DF, DFDC	CNN	XceptionNet	Transfer learning: With transfer learning 86.49%, without transfer learning 79.62%
[10]	Celeb-DF-FaceForensics++ (c23)	CNN	YOLO-CNN-XGBoost	90.62% AUC, 90.73% accuracy, 93.53% specificity, 85.39% sensitivity, 85.39% recall, 87.36% precision, and 86.36% F1 score
[62]	DFO, FSh, CDF, and DFDC	ResNet-18 and MS-TCN	Semantic irregularities	82.4% for CDF, 73.5% for DFDC, 97.1% for FSh, and 97.6% for DFo datasets.
[63]	FF++, DFDC, and CDF	CNN	Multi-attentional framework	97.60% for FF++, 67.44% for CDF and 0.1679 Logloss for DFDC.
[64]	DFD, CDF, and FF++	NN and CNN	Multi-feature fusion	99.73% for FF++, 92.53% for DFD, and 75.07% for CDF dataset.
[65]	DFDC	NN and CNN	NN compression	93.9% for DFDC dataset.
[66]	J48	TIMIT-DF, DFD, and CDF	Feature fusion	94.21% for TIMIT-DF, 96.36% for DFD, and 94.17% for CDF.
[67]	3D CNN	FF++, TIMIT HQ, TIMIT LQ, DFDC-pre, and CDF	Channel transformation	99.83% for FF++, 99.28% for TIMIT HQ, 99.60% for TIMIT LQ, 93.98% for DFDC-pre, and 98.07% for CDF dataset.

## Data Availability

Not applicable.
